# ‘We are not the ones to blame’. Gamblers’ and providers’ appraisal of self-exclusion in Germany

**DOI:** 10.1186/s12889-023-15117-9

**Published:** 2023-02-14

**Authors:** Ludwig Kraus, Andreas Bickl, Lucia Sedlacek, Larissa Schwarzkopf, Jenny Cisneros Örnberg, Johanna K. Loy

**Affiliations:** 1 IFT Institut für Therapieforschung, Centre for Mental Health and Addiction Research, Leopoldstraße 175, 80804 Munich, Germany; 2 Department of Public Health Sciences, Centre for Social Research on Alcohol and Drugs, Stockholm University, Stockholm, Sweden; 3 Institute of Psychology, ELTE, Eötvös Loránd University, Budapest, Hungary

**Keywords:** Gambling, Self-exclusion, Legal regulations, Interviews, Conflict of interest

## Abstract

**Background:**

Given low utilization by individuals experiencing gambling problems the potential of self-exclusion (SE) might be not fully exploited in Germany. This paper aims to gain insight into different actors’ perceptions and reflections on the problems and difficulties in the process of self-exclusion to delineate which specific attitudes hamper a successful implementation of SE.

**Methods:**

13 individual and four group interviews with individuals experiencing gambling problems and governmental or commercial gambling providers were examined. A Grounded Theory Approach was used to portray the opinions of these different actors on existing regulations of SE and to delineate potentially diverging interests between the distinct groups.

**Results:**

The interviewees agreed on the usefulness of SE and consented that it is important to early recognize individuals experiencing gambling problems. They also considered the present practice insufficient but for different reasons. Individuals experiencing gambling problems and providers particularly disagreed on addressing individuals experiencing gambling problems. While individuals experiencing gambling problems stated that they had hardly ever been approached, providers argued that help offers were mostly rejected. Especially commercial providers also regarded insufficient German language skills and rapid fluctuation of guests as strong barriers to approaching individuals experiencing gambling problems. Interviewees from governmental venues furthermore suspected that commercial providers took addressing individuals experiencing gambling problems less seriously.

**Conclusion:**

Our results emphasize the dilemma of conflicting interests in both individuals experiencing gambling problems and providers. Rather than acting against the economic interests of employers, venue staff blame individuals experiencing gambling problems for lack of problem recognition. Conversely, individuals experiencing gambling problems blame the providers for not offering help. To address individuals experiencing gambling problems appropriate staff training is required, and SE regulations need to be controlled by an independent body rather than by the providers themselves.

## Introduction

Gambling is considered a high-risk entertainment activity enabling feelings of euphoria and a sense of achievement. Excessive use can lead to gambling disorder (GD), which is accompanied by psychological, social, and financial problems [1]. Moreover, negative consequences for individuals affected such as debt, unemployment, or treatment must be borne by public resourses [2]. To minimise gambling-related harm to gamblers and society, protective behavioural strategies are recommended [3]. One possible measure is the option for gamblers to self-exclude from certain venues, particular games, or gambling at all [4].

Voluntary self-exclusion (SE) has a two-way preventive function. As a precautionary measure, it can protect individuals experiencing gambling problems from developing GD and simultaneously prevent them from relapsing or further gambling-related harm. There is evidence for a variety of benefits of SE including a reduction in both psychological strain and interpersonal difficulties, increased work performance, enhanced quality of life, and financial improvement [5–9]. Previous research, however, identified structural barriers preventing comprehensive utilization of such strategies [10–12]. Moreover, studies indicated low compliance of providers regarding information of customers about SE options [13, 14].

Despite being associated with positive effects, utilization of self-exclusion among individuals experiencing gambling problems remains low [7, 15, 16]. In Germany, data on the uptake of exclusion options are only available for casino gambling where a nationwide documentation system exists (OASIS). Corresponding data from 2020 suggest, that in total 62,000 persons were self-excluded. Based on an estimated 1.3 million individuals with pathological gambling behaviour and 4.5 million persons with problem or pathological gambling behaviour [17], this corresponds to 4.8% and 1.4% of gamblers who self-excluded from gambling, respectively. These numbers refer to SE from governmental providers in all federal states and commercial providers in Hessia only as the other federal states were not integrated into the exclusion system and did not provide figures on SE. In addition, at the time of data collection, with the exception of one federal state (Schleswig-Holstein) online gambling was illegal in Germany and therefore regulations of self-exclusion for online gambling did not exist. Nevertheless, residents of other regions may have gambled online or self-excluded from online gambling activities.

Legal obligations to implement and execute preventive measures such as providing and supporting problem gamblers to self-exclude from gambling restrain providers’ financial interests as individuals experiencing gambling problems represent a loyal and profitable customer group [18]. It is estimated that approximately 80% of turnover stems from about 20% of all customers with the majority of them classifying for GD [13]. Hence, lobbying, insufficient compliance of providers, and gaps in the supervision of excluded gamblers seem to hamper the appropriate implementation of protective behavioural strategies [14].

Against this background, the current paper aims to delineate diverging interests between individuals experiencing gambling problems as well as governmental and commercial providers by analysing individual and group interview data from the VeSpA interview study (Verbesserung der Spielersperren-Ausgestaltung - Improving Self-Exclusion Regulations). Based on a previously published qualitative analysis of the VeSpA interview data [19], the opinions of individuals experiencing gambling problems as well as governmental and commercial gambling providers regarding SE and their views on the existing regulations at the time of the interviews were compared. While the first publication of the study focused specifically on the need and possibilities for improving SE to achieve effective gambler protection, the aim of the present analysis is on examining differences in the stakeholders’ perceptions and reflections on the problems and difficulties in the process of self-exclusion to delineate which attitudes hamper successful implementation of SE.

## Methods

### Background on self-exclusion framework in Germany

Since 2008 gambling in Germany is organized by the State Treaty on Gambling (STG) and federal state-specific implementation laws, regulating state-run (governmental) and licensed (commercial) gambling. This resulted in regulations for the initiation and monitoring of SE for governmental gambling (casinos) but excluded commercial gambling providers (gambling halls) [20]. While, at the time of data collection, for the former, there was a nationwide exclusion system which could be applied for at any casino in Germany or by post, the regulations for gambling halls differed from federal state to federal state. The situation was further complicated by the fact that commercial providers were not explicitly obliged to offer SE, which led to inconsistencies in the formulation and reach of regulations between the federal states. While only two federal states offered comprehensive SE options for any land-based commercial provider (Hessia, Rhineland-Palatinate), in some others (e.g., Bavaria, Lower Saxony, Saxony, Thuringia, Saxony) SE had to be applied for in each venue separately. Consequently, effective access prevention could not be granted as excluded individuals experiencing gambling problems could not be identified across both governmental and commercial providers nationwide [21]. Also, the process required by the commercial providers was more complicated compared to governmental ones. In many regions individuals experiencing gambling problems had to make an appointment with the person commissioned for SE and a personal appearance at the gambling hall was required. Although a clearance certificate was required for revoking SE, binding criteria for acceptance were not defined making the process arbitrary and solely dependent on the providers’ decision. Regarding exclusion duration, only a minimum of one year was specified.

On July 1, 2021, the 4th revision of the STG [22] came into force, which reformed among others SE by harmonizing and extending the former nation-wide register for self-excluders from governmental venues to those who self-exclude from commercial venues. Accordingly, the revocation process and the self-exclusion’s duration have also been simplified. Revocation can be initiated by applying to governmental authorities; the minimum blocking duration has been reduced to three months. This study was conducted before the current version of the STG became effective.

### Data and design

The VeSpA interview study aimed at gaining qualitative information on gambling SE in Germany from various stakeholders (gamblers, relatives, governmental and commercial gambling providers, staff members of the Society for Gambler protection and Prevention (Gesellschaft für Spielerschutz und Prävention, (GSP)) and was conducted between December 2018 and July 2019. The focus of the interviews was on rules, conditions of self-exclusion, personal experiences, and perceived barriers. Details of the study design have been published elsewhere [19].

### Recruitment of study participants

Flyers to recruit gamblers were displayed in venues and outpatient addiction care centres or handed out by cooperation partners. The majority of the participants were recruited by distributing the flyers in Bavarian Gambling Addiction Care facilities. In addition, interviewees were recruited online via the social media platform Facebook, web pages for gambling addiction information and counselling as well as postings in gambling addiction self-help forums. Interviewees were reimbursed for travel expenses and received an incentive in the form of a € 20 voucher. For the interviews with Bavarian casinos’ gambler protection officers’ appointments were made for two group interviews. The contact for two group interviews with the employees of the gambling halls was arranged by the GSP, that advises gambling operators on player protection measures. The aim of the group interviews was to discuss and exchange different perspectives on self-exclusion regulations and implementation procedures [19].

### Participants

The initial VeSpA sample consisted of 26 individual and 6 group interviews. To mirror the perspective of individuals experiencing gambling problems, staff members of commercial gambling halls and those of commercial casinos on the process of SE, a subsample of 13 individual and four group interviews was selected for the present analysis. This sample of interested participants comprised (1) 12 individual interviews with gamblers, (2) two group interviews with managers from different casinos who were in charge of gambler protection at their casino (each with four participants), and (3) one individual and two-group interviews with staff of different commercial gambling halls.

### Interviews

The interviews were based on semi-structured open questions covering the current legal regulations of SE from gambling in Germany, their implementation, and options for optimization. The topics covered encompassed among others the design of SE (type of gambling, valid for single or all venues, length, revocation), aspects for improvement, and the use of help services. The interviews were conducted face-to-face or by phone and were recorded; the duration of the interviews varied between 25 and 120 min.

### Data analysis

**Fig. 1:**
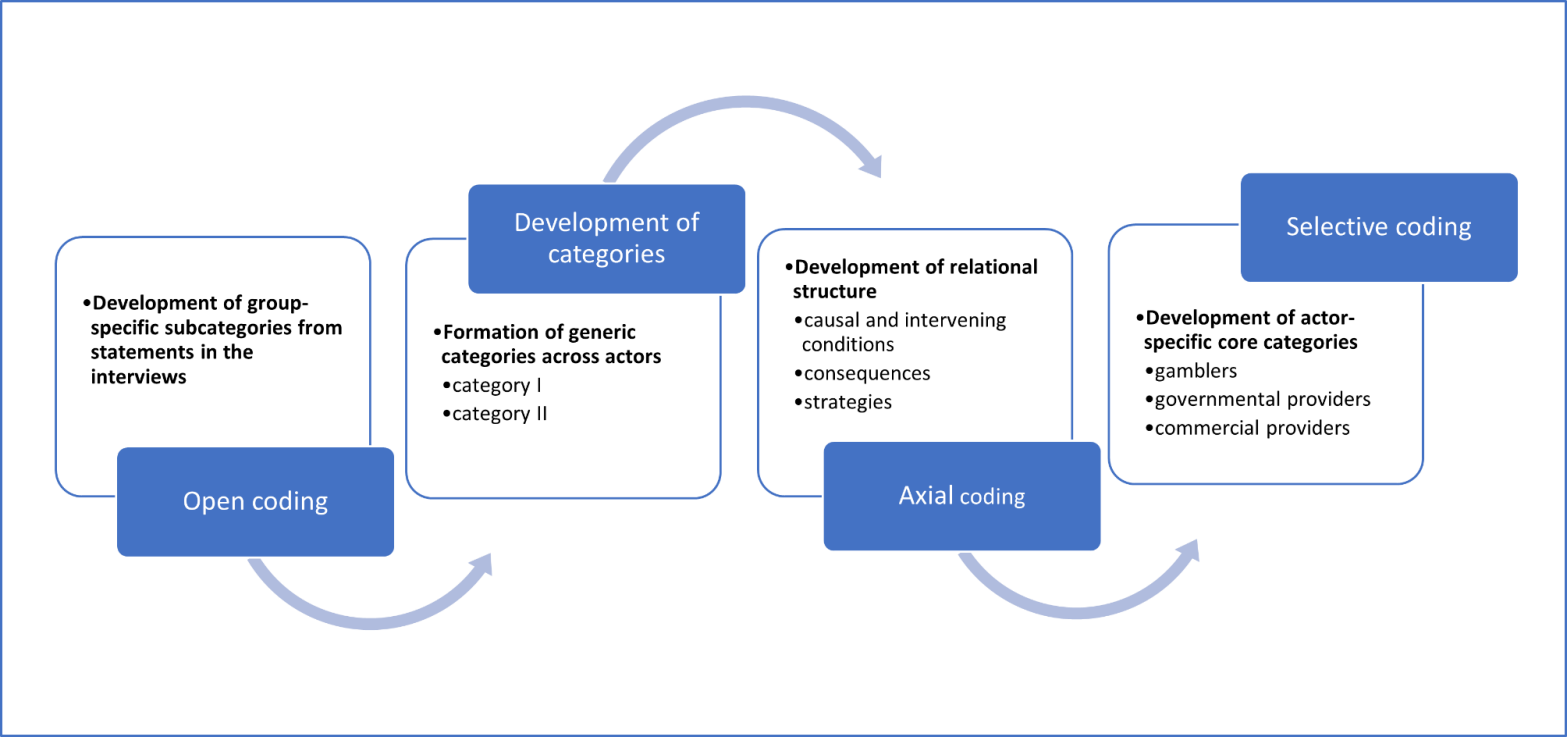
Analytical process based on grounded theory

For elaborating and contrasting the interests of the different actors, a grounded theory approach to conceptualize the data was used [23, 24]. The comparative approach aimed to inductively identify statements of the actors regarding their views on SE and to condense these until core categories could be derived. The data analysis followed a stepwise procedure. In the first step of open coding, the raw statements of all participants in the subsample were coded concerning the terminology they were using. From these open codes, several group-specific subcategories were identified. Superior generic categories across all interviews were generated. In the second step (axial coding), the categories were grouped and set in relation to each other. This grouping process was oriented towards causal as well as intervening conditions, strategies, and consequences in dealing with SE, which are brought into a relational structure regarding our research question. Finally (selective coding), the subcategories and categories were integrated and condensed into one distinct core category for each of the three actors. The core categories underlie the statements and actions of the actors and should make their actions ‘understandable’ (Fig. 1).

All interviews were conducted in German – irrespective of the interviewees’ native language. The interviews were fully transcribed with a conversation analytic transcription system (Gesprächsanalytisches Transkriptionssystem; GAT). The coding was constructed using the MAXQDA 2018 software [25]. Each interview was coded separately and individual statements were assigned to codes by three independent evaluators (JL, AB, LuS). Throughout the analytical process, the three analysts met regularly, discussed the details of their findings and confirmed their agreement over the emerging (core) categories. In the individual interviews, the real names were replaced by changed first names, in the group interviews there was no differentiation of speakers and therefore no names were used. The reported quotes were translated by a native English speaker.

### Ethics

Study participants were informed about background, objectives, procedure, and general conditions of the study before providing written informed consent. The VeSpA study received ethical approval from the Ethics Commission of the German Psychological Association (DGPs) (Az: AH 082018). All methods were performed in accordance with the Declaration of Helsinki [26].

## Results

Among the twelve individuals with gambling problems, ten were male (83%) and nine reported an active ban from gambling (75%). All governmental provider interviewees were male and among the interviewees from commercial providers four were female and five were male. We identified two categories for the three actors and one core category for each actor (Table 1).

**Table 1:** Identified categories and core categories

Categories			
Early Recognition/Active Addressing (category I)		Length and Revocation (category II)	
**Core categories**			
**Gamblers**	**Governmental providers**		**Commercial providers**
Self-exclusion has benefits, but there are limitations	The underlying idea of self-exclusion is positive, but it does not work		If SE worked as intended, it would be an appropriate measure as people with gambling problems should be prevented from gambling

### Individuals experiencing gambling problems

Several gamblers stated that **self-exclusion had benefits, but that there were limitations** (core category gamblers). Basically, there was a positive general attitude:

*“I recommend them to everyone with whom I talk about it.” (Alfred)*

*“… and I don’t know, I would of course recommend everyone to use self-exclusion.” (Werner)*

Nevertheless, the interviewees expressed limitations of the current regulations, which might help explain the low rates of people with gambling problems using self-exclusion. Alfred emphasized that a good implementation of the system was important and criticised the current complexity. Carl and Peter expressed:

*“But (…) no one does it (Pos. 91–92), I am not banned right now for the main reason that it doesn’t work.” (Carl)*

*“In principle, you can then continue to play in the same or in other gambling venues. So there is no control with ID cards or anything else. So it doesn’t really help at all.” (Peter)*

The main reasons for their criticism were the possibility of shifting to other gambling venues, the high effort necessary for getting excluded in each hall separately, the lack of ID controls when entering the place, and the low level of utilisation in general.

There was high agreement among the interviewees regarding **Early recognition and actively addressing gamblers** (category I). The interviewees considered this as necessary for the implementation of SE. In their opinion, addressing noticeable gambling behaviour did not take place. They blamed gambling providers and accused them of not being interested in protecting gamblers and of not giving enough information on self-exclusion:

*“…. in my career of gambling, no one has ever asked for an ID or a thing. No matter how much money I gambled. No matter how long I was in there. As if this interested the staff. Ah, it’s just about / So I will say it this way now. ‘Oh, that idiot is back again’, but never in the way of ‘He needs help’.” (Carl)*

*“That doesn’t happen. It’s like when I get plastered. Then no one approaches me about it either, yes?” (Alfred)*

*“But as I said, no one has ever approached me in my regular play hall. So, I didn’t know… I honestly didn’t deal with it now because I actually thought that there was no such thing that I could use.” (Rainer)*

*“I would certainly have taken advantage of that in my times of crisis. [if the offer had been available there with flyers]. I would certainly have made use of it.” (Margot)*

From the individuals experiencing gambling problems’ point of view, there is a need for being addressed and supported in considering SE. They would not take advantage of SE or might take advantage of it too late if there was no addressing by third parties. Especially for people with emerging gambling problems, active addressing by the staff was considered important. Otherwise, due to missing problem recognition, individuals experiencing gambling problems would not resort to a ban, even a temporary one. In this context, several gamblers pointed to the severe negative consequences of gambling. They clearly stated, if they had been addressed by staff in the casino, it would have helped them in time, might have prevented their problems from getting worse, and also might have prevented the accumulation of debt.

In addition to a lack of interest in gambler protection, the conflict of providers between gambler protection – a legal obligation for staff members – and creating a cosy atmosphere, was considered a reason for the deficiency in addressing gamblers:

*“That would lead the business model completely ad absurdum. So, either it doesn’t take place, or it takes place with the stipulation: Don’t take this so seriously. Bring another coffee instead. Yeah. So that the people feel comfortable.” (Alfred)*

Similarly, Werner expressed that addressing of gamblers by the staff would make sense, but also clarifies that complying with this legal obligation means risking problems with their superiors:

*“That also would be something, I think it would be good to say to these service staff: ‘Listen, if someone comes in who has been here with you for years and you see that he goes in there and gambles away all his money, why don’t you talk to him about it, you should say for example: ‘Hey, you, listen, you can get yourself blocked here. Then you don’t need to come in here anymore’. But I’ve already experienced that she says: ‘You know what? Then I’ll be out of a job, because then my boss will come and say, ‘You, why do you encourage people [to self-exclude], who always bring money?’ To get blocked, they are not allowed to do that.” (Werner)*

The gamblers not only reported the missing of white nudges (positive incentives) facilitating the self-exclusion, despite the obligation of the providers to actively address gamblers [27], but even mentioned nudges to disrupt self-exclusion: Peter stated that employees even tried to talk him out of the idea of excluding himself:

*“Yes. This person represented 99% of his employer’s interests (I: laughs) and more or less tried to get me to play a bit more carefully and consciously, and an exclusion would not be necessary and that [SE] is hardly ever done and would be so unusual. So, in principle, it was a farce.” (Peter)*

**Length and revocation of self-exclusion** (category II) were discussed with great ambivalence and partly contradictory. Despite arguing once in favour of one year as a manageable length of exclusion resulting in lower barriers for self-banning, Carl and Rainer subsequently voted for unlimited exclusion:

*“So, it needs to be lifelong and then explained somewhere, well maybe one should at some point in the future be able to reverse it (…). But limiting it to one year doesn’t make any sense, because after one year I will certainly not yet be healed from the gambling. (…) I don’t want to exclude them, the opportunities to repeal. But definitely unlimited.“ (Carl)*

*“I would certainly have taken a longer period, simply in these phases where I want to stop, I was always very radical. So I would probably / maybe even have said “for the rest of my life”. (Rainer)*

For people with GD a temporary exclusion was seen as too short, but it was acknowledged as a helpful option in an early career. On the other hand, an unlimited exclusion with high or even insuperable barriers for revocation was considered a deterrent, too restrictive, and a reason for low rates of utilisation. The interviewees endorsed the possibility to choose between an unlimited exclusion and one which is limited to some time between two or up to five years (Alfred) or one year (e.g., Carl, Werner). They stated that, for successful implementation of SE, the length should be adapted to the extent of problem severity with the possibility for individuals experiencing gambling problems to choose between different periods. Alfred recommended a minimum length of exclusion. He repeatedly emphasized that a ban should at least last more than one year, as he felt that one year was too short to be helpful.

Options of SE revocation were further discussed in detail. In case of temporary exclusion, no automatic expiration but an active revocation was proposed. A renewal for the same period as the initial SE should apply if the revocation was not accurately timed. The interviewees supported the idea of realistic barriers for revocation, e.g., demonstration of treatment or individualized advice for individuals experiencing gambling problems. The current regulations, however, were criticised for not being practicable as they do not seem to offer a realistic possibility to revoke banning (for a summary see Table 2).

**Table 2:** Summary of arguments and concerns by actors

Gamblers	Governmental providers	Commercial providers
***SE has benefits but there are limitations*** - Complexity of the system - High effort of the exclusion process - No ID controls - Possibility of shifting to other venues ***Early recognition and actively addressing gamblers*** - Lack of interest in gambler protection by providers - Lack of information on self-exclusion - Lack of support and actively addressing by providers - Conflict of interests/role for providers ***Length and revocation of SE*** - Duration of offered temporary exclusion too short - Necessity of revocation possibilities with realistic barriers after the minimum length	***The underlying idea of self-exclusion is positive, but it does not work*** - Possibilities of shifting to governmental or online gambling for excluded gamblers - Necessity of tackling causes for gambling addiction instead of solely excluding gamblers - Lack of problem recognition by gamblers ***Early recognition and actively addressing gamblers.*** - Gamblers’ disapproval of being actively addressed ◊ Lying, denying and downplaying by gamblers - Casino visits as a hobby and social meeting points - Problems mainly caused by gambling halls, illegal and online gambling (“hypocrisy of the staff members in gambling halls”) ***Length and revocation of SE*** - Heterogenous views on length and revocation of SE - Necessity of individualized regulations	***If SE worked as intended, it would be an appropriate measure as people with gambling problems should be prevented from gambling*** - Necessity of additional psychological support in addition to SE ***Early recognition and actively addressing gamblers*** - Providers already take sufficient responsibility - Training (e.g., for actively addressing) perceived important and helpful - Lack of problem recognition by gamblers - Gamblers’ disapproval of being actively addressed ◊ no chance for staff to be successful in addressing gamblers - Gamblers’ reactions on being actively addressed: aggression, anger, denial - Language barriers for staff and gamblers ***Length and revocation of SE*** - Necessity of individualized regulations regarding duration of SE

### Governmental providers

In the group interviews with staff members from governmentally run casinos the following core category could be identified: **The underlying idea of self-exclusion is positive, but it does not work** (core category governmental providers):

*“[self-exclusion] is definitely a sensible measure”. (I1, group interview)*

*“But it’s not very successful”. (I2, group interview)*

Governmental providers considered SE in principle a reasonable measure for people with gambling problems in need of support and protection. The interviewees emphasized that they provided support and did all that they could do to have a working self-exclusion program and to protect vulnerable individuals experiencing gambling problems. The effectiveness of SE, however, would be very restricted due to evasion possibilities like gambling somewhere else, e.g., online gambling, illegal gambling, gambling in gambling halls or abroad. They secondly argued that SE was a questionable approach because it tackled gambling problems only symptomatically. The causes of pathological gambling were not adequately accounted for. Furthermore, it was stated that individuals experiencing gambling problems often lacked problem recognition and hence did not perceive SE as an option. This would hamper the effective addressing of gamblers.

The lack of problem recognition by gamblers is closely connected with the first category: **Early recognition and actively addressing gamblers.** The interviewees emphasized being sensitized for problem indicators such as stress, sweating, obvious negative behaviour, and shaking or strongly changed betting levels – with increased money spent seen as a potential indicator for loss of control and greatly reduced expenses seen as a potential indicator for financial problems. Of significant importance was the change and not the level of expenses per se. One participant recollected situations in which sensibly and responsible actions had been taken by addressing people with gambling problems:

*“But when it starts going in that direction, one has to step-in in time. When he ends up only playing with the ones where he used to play with fifties, he probably has a financial problem (…). We deal with that responsibly, very responsibly even.“ (group interview)*

The interviewees expressed the opinion that high expenses were not a sign of problem behaviour per se as they might mirror an expensive hobby with the possibility of winning:

*“Everyone has that right. The other person has a motorcycle, the other person has / who knows what? The other person says ‘Okay, I will play every month a specific amount’. The other person says: ‘I will put the amount away for’, I don’t know, ‘a car’ and spends €30,000 or €40,000 in one go for a car. And that’s the way it is. He says: ‘Okay. I have this hobby’. Or, who knows ‘I will go every month’ I don’t know, ‘one, two, three, four times to a casino’ and then it is just that same amount that is just gone as well. But potentially here, he also has the chance that he wins something (…).” (group interview)*

The interviewees also emphasized that addressing individuals experiencing gambling problems needed to happen sensitively. They were not approached in front of others, staff talked to them tentatively, had some small talk, and reported what they had noticed. Staff members were said to try to make the customer understand that s/he ought to seek help, and not to harass gamblers by directly suggesting self-exclusion. As reactions when being addressed, individuals experiencing gambling problems would often lie, deny, or trivialize their situation.

The interviewees also stressed differences between online gambling and commercial gambling venues, affirming that their staff sanctimoniously claimed to care for vulnerable individuals experiencing gambling problems, while in reality, they did not at all (belonging to category I).

*“(…) The hypocrisy of the staff members in gambling halls... They say (…) blah blah blah, but no one does it, to actually enforce an exclusion. " (group interview)*

In contrast, governmentally run casinos were not only seen as gambling venues but also as social meeting points, with a possibility of having fun and excitement with little money and where one got an additional cultural program such as live music.

*“(…) With a fifty I can hold myself (…) above water for one and a half hours (…). Then I have had a bit of excitement, I have / when I have friends with me, simply it was a meeting too, many come, especially older semesters, who know: when I go to the casino, I meet some guys (…). They are these meeting points too and in Bavaria, there is no compulsion to play.“ (group interview)*

The second category **length and revocation of SE** was not discussed concordantly among the gambling operators. Different lengths depending on problem severity were regarded to be important by all interviewees. Also, barriers for revocation were homogenously considered far too high. An unlimited exclusion was generally refused for its deterrent effect and an ability to choose between different lengths of exclusion was considered a means to increase its acceptance and consequently utilization. Altogether, governmental providers preferred individuals experiencing gambling problems to stay away for a while, only, to ‘have a little think about it’ and to take responsibility. One participant said:

*“When someone is wise enough to say: ‘I am going this way’* [of choosing self-exclusion] *and they are serious about it, then one should offer them a perspective, so they don’t say ‘that way* [visiting casinos again] *is blocked for me forever’.” (group interview)*

Regarding the length of exclusion, a minimum of one year was preferred by the interviewees as individualized regulations would better meet the needs of individuals experiencing gambling problems (for a summary see Table 2).

### Commercial providers

In the responses from interviews with the staff members of the commercial gambling venues the following core category could be identified: **If SE worked as intended, it would be an appropriate measure as people with gambling problems should be prevented from gambling**. For the implementation of SE, the interviewees had several concerns. In addition to the low utilization, SE was not considered sufficient to overcome pathological gambling. It was argued that additional psychological support would be necessary.

*“So with this exclusion which is requested online, so if he wants to do that, he can maybe fill out a document online, but I still think it’s important that the gambler who wants to do that also talks to someone who is trained to do that, to see if he has, I don’t know (…) Whether only the exclusion is useful or whether therapy would be better and/or both together logically or / Yes.“ (group interview)*

Regarding the first category **Early recognition and actively addressing gamblers**, participants emphasized the need to support individuals experiencing gambling problems by actively addressing people showing problem behaviour. They noted that talking to the guests was quite important:

*“(…) we can also save someone with it. We can offer help.“*

One of the participants referred to the company’s guidelines:

*“The company says that they simply don’t want to make money with it or not earn any money there and not make any revenue with pathological gamblers, because it is a disease and that doesn’t need to be encouraged.“ (group interview)*

Staff members held different opinions on the identification of problem behaviour (belonging to category I). Some remarked that identification through the amount of money spent was difficult without knowing details on the person’s financial situation. Furthermore, the location of some gambling halls (e.g., at railway stations) is linked with a high fluctuation of guests (‘walk-in customers’) rendering the recognition and addressing of individuals experiencing gambling problems difficult. Most interviewees affirmed the existence of visible signs of problematic or pathological gambling like aggressive or nervous behaviour, testiness, or changed expenditure. They described that getting to know the customers personally was important for recognition and addressing problems as a trusting relationship was seen as a necessary precondition:

*“When you don’t know < the person>, of course, you can’t tell him/her: ‘get yourself banned’, … Maybe he has on that day a bad day. Maybe s/he is stressed, went in, gambles, is nervous about other things (…). No, you can only tell him/her: ‘Take a break today. Go home, come/try again tomorrow’.“ (group interview)*

The training they had received for these tasks was perceived as helpful:

*“Yes. This is good for me. I am happy because I have a safe feeling and that makes more fun at work. (…) So early detection, this is best with us and naming problems clearly and (…) We learned that.“ (group interview)*

The staff members described how they proceeded when addressing customers because of striking gambling behaviour (belonging to category I). When they recognized salience, they discussed it with the team and took notes in their early recognition software, a computer program for tracking problematic gambling behaviour. For approaching targeted guests, it was seen as important to choose an adequate situation, a friendly tone, to be very discrete, and not to talk in front of others. A common way was the suggestion to go for a cigarette and to create a trusting and confident atmosphere. One participant emphasized the necessity of using I-messages:

*“Also don’t say to him: ‘Yes, hey. Hey, you have a gambling problem. Do something with it’. We also should not say something like ‘you are addicted’ (…). You better say ‘I noticed, you come quite often…’or something like that.” (group interview)*

Another interviewee summarized:

*“So, I managed that several times, but not always. With some people you can try what you want, it doesn’t matter.“ (group interview)*

Language barriers on both sides were mentioned as aggravating circumstances (belonging to category I): On the one hand, many customers did not speak German, or the languages staff members could speak and understand. On the other hand, many staff members did not speak German fluently and mentioned their difficulties regarding addressing individuals experiencing gambling problems and proper descriptions of opportunities for help.

The staff members also reported their experiences with customers’ reactions when raising the issue of problem gambling (belonging to category 1). According to the interviewees, people addressed usually felt ashamed, rejected, and did not want to be disturbed because they were focused on the game. They often became angry, dismissive, did not like the staff to intervene, and said that they neither needed nor wanted to self-exclude. Most individuals experiencing gambling problems had a lack of problem recognition and rejected any help offers. Only very few reacted positively and considered to self-exclude. In these cases, staff members encouraged guests in their decision to exclude themselves and supported the implementation of the exclusion process.

The second category was **Length and revocation of self-exclusion**: In all interviews, the view was held that problem gambling was chronic and led to serious negative consequences. Staff members distinguished between individuals with high and low problem severity and those without problems. For the former cases, they favoured unlimited self-exclusion which should not be revocable:

*“It’s better not to repeal when he’s addicted (…). Yes, because then he comes back to the same problems, maybe even more, and then these suicides happen. So, depressions can come, come in addition, definitely. So*, [an exclusion that is not revocable] *would be better.“ (group interview)*

Under the regulations being valid when the study was conducted, commercial gambling halls, next to unlimited exclusion, offered exclusion of 3, 6, and 9 months. The shortest period was regarded as ineffective and according to the providers would not be used. Six- and nine-month options, however, were seen positive and should be maintained (for a summary see Table 2).

## Discussion

The groups of interviewees (gamblers, governmental and commercial providers) concurred on the numerous negative effects of GD as well as the basic idea of SE as a protective measure, although individuals experiencing gambling problems considered SE more effective than providers. The respondents also agreed on the limitations of SE. It was acknowledged that SE is not sufficient to overcome gambling problems, as it addresses the symptoms rather than the underlying problems of addiction. SE was also considered ineffective as long as numerous alternative options to gamble existed. The interviewees consented to the importance of early recognition of individuals experiencing gambling problems and their active addressing to alleviate or even prevent negative effects of gambling such as worsening of symptoms and high monetary expenditures. There was also consent regarding the criticism of the ‘regulations for revocation of SE’. The requirements to revoke SE were said to be too strict and the de facto unlimited exclusion was considered the main reason for discouraging individuals experiencing gambling problems from initiating SE. It was suggested to introduce individual exclusion periods that might help to mitigate problem severity, and to make therapy or counselling during exclusion mandatory.

Dissents emerged regarding ‘addressing individuals experiencing gambling problems’ between individuals experiencing gambling problems and staff in commercial gambling venues and governmental casinos. The interviewed individuals experiencing gambling problems stated that they were hardly ever approached and suspected competing interests as the main reason. Contrary, the providers assured to approach individuals experiencing gambling problems but reported that offers of SE were rejected in most cases. Individuals experiencing gambling problems’ lack of problem recognition’ was argued to be the main reason for not initiating SE. Interviewees from commercial gambling providers reported to not only train staff in early recognition of problem gambling but to also use software for tracking gambling behaviour. They further considered insufficient German language skills as strong barriers to approaching individuals experiencing gambling problems adequately. Recognising problem behaviour was considered challenging because of the rapid fluctuation of guests in commercial gambling venues. Finally, the interviewees from governmental gambling casinos suspected that commercial gambling venues took addressing of problem gamblers less seriously.

There was no consensus on an ideal minimum duration of SE neither within nor between groups. The interviewed governmental employees stated that the length should consider individual problem severity and provide sufficient time for individuals experiencing gambling problems to regain control over their behaviour. The opinions of staff from commercial providers on length and revocation of SE were strongly built upon the perception that individuals experiencing gambling problems cannot recover at all and need to be banned for a lifetime.

The results of the present analysis indicate that the potential of SE is not fully exploited, and utilization is low, even if all participants described SE as helpful and supportive for approaching problem gambling. In a study by Ladouceur [12], a large proportion of individuals experiencing gambling problems stated that because of the exclusion they had stopped gambling completely. Studies also could show that the ban leads to significantly reduced urges to gamble and increased perception of control [8]. Nevertheless, prior studies – particularly of German origin – demonstrated that vulnerable individuals experiencing gambling problems are rarely addressed especially in gambling halls [14, 21, 28], and that providers exercise little or no influence on those unwilling to recognise and counteract their problem gambling behaviour [11]. Furthermore, lack of staff training in identifying individuals experiencing gambling problems, particularly in crowded venues, and competing interests for venues reliant on gambling revenue were recognized as main barriers [11].

Although low utilization rates might be explained by language barriers on both sides (gamblers and staff members), the staff’s attempts to explain low utilization unanimously by individuals experiencing gambling problems’ lack of problem recognition and their reluctance in disrupting the flow of gambling seem to be justifications rather than rational considerations. The gap between the moral pressure on staff to put gambler protection regulations into force and the everyday routine in gambling halls and casinos with sometimes high customer fluctuation, little room for interventions, and the inherent inability of individuals experiencing gambling problems to reflect their gambling behaviour create strong cognitive dissonance. Rather than acting against the economic interests of their employers and acknowledging the lack of problem recognition as part of the problem, staff members resolve the issue by blaming individuals experiencing gambling problems for their ‘ignorance’ or the environmental circumstances. Similarly, individuals experiencing gambling problems, particularly once they are in trouble or reflect on their gambling from a distance, blame the providers for not having addressed their problems and not having offered help when they presumably had needed it most.

Based on the opinions expressed by both gamblers and providers some recommendations can be derived that may increase the uptake of SE and enhance its effectiveness. The implementation of mandatory self-checks with guided questions and feedback on the individual’s gambling behaviour could be useful in terms of early recognition, rising problem awareness, and increasing acceptance of support offers [29, 30]. Such self-checks could also mitigate the occurrence of cognitive dissonance and the need to shift responsibility. The need for additional support as an intermediate step becomes evident by acknowledging that most individuals experiencing gambling problems lack introspection regarding their gambling behaviour [31]. This renders interventions at various stages of the gambling activity including active addressing necessary. Similar to alcohol compliance checks conducted by law enforcement agencies [32], an independent body monitoring governmental and commercial providers’ compliance to gambler protection regulations including but not limited to early recognition activities may be suitable for circumventing conflicts of interest between employers and staff [13]. Another option is the tracking of gambling behaviour with personalized feedback in case of high-risk gambling [33, 34]. The possibility of individualized duration with a minimum length of the blocking would enhance gamblers’ independence in decision making. Any gambling ban should actively be revoked by the individuals experiencing gambling problems in contact and consultation with addiction help services to reduce relapse in not yet stable individuals [35]. As a single strategy SE cannot solve GD but needs to be accompanied by counselling or comprehensive therapeutic approaches [4].

The positions of the interviewees and their suggestions anticipated some regulations of the 4th STG which has come into force on July 1, 2021. In particular, preventive measures such as deposit limits for online gambling, ban of parallel gambling on two or more gambling machines, electronic early detection systems, and the establishment of a central exclusion register covering all land-based gambling forms, online casinos, and online sports betting have been introduced to centralise the previous nationally heterogeneous regulations on SE. Particularly, the need for a central register had been acknowledged by all interviewees [19]. These measures are accompanied by the – so far pending – implementation of a Joint Gambling Authority, which is supposed to supervise the central tasks of gambling supervision at the federal level.

Nevertheless, with the present more individualized approach to reducing gambling harm, Germany’s SE program may be considered rather ineffective. According to a recent study comparing public health implications of SE programs in seven high-income countries or states including Germany, policy approaches intend to balance attempts in reducing gambling problems and the industry’s interest in increasing gambling revenue [36]. Thus, the reach and extent of enforcing SE vary depending on the respective priority setting. The authors however concluded that a public health approach is more suited to strengthen the effectiveness of SE programs than transferring the responsibility to the individual gamblers alone.

### Limitations

Several potential limitations are related to this analysis. First, the qualitative nature of our study renders generalization difficult. It gained, however, insight into the individual perspectives of various involved parties. In fact, descriptions of the current implementation of the legal regulations differed widely between and within the interviewed groups. Second, some findings were obtained from group interviews which compared to the individual interviews were less in-depth and detailed. In addition, biases resulting from tendencies to provide socially acceptable answers, or, particularly in the group interviews, succumb to group-specific norms cannot be ruled out. Nevertheless, the group interviews resulted in the reporting of a wider range of experiences and provided insight into intra-group differences that may help revise the legal regulations. Third, individuals experiencing gambling problems opinions were influenced by their own experiences or second-hand reports. These, however, were highly comparable across all gamblers interviewed and there was no gambler in the sample without any first- or second-hand reports.

## Conclusion

Our findings emphasize the dilemma between the need for help and the desire for autonomy among individuals experiencing gambling problems and point to venue staff’s barriers in addressing problem behaviour by illuminating competing interests. The emerging cognitive dissonance in individuals experiencing gambling problems for not being able to solve their gambling problems and in staff for failing to address these problems adequately, result in cognitive coping strategies. These include but are not limited to justifications such as the lack of emphatic and active interventions of venue staff expressed by individuals experiencing gambling problems, and staff’s irrational reproach that individuals experiencing gambling problems lack problem recognition. Need-based approaches considering the individuals experiencing gambling problems’ personal circumstances combined with standardised supervision of venue staff might be a suitable strategy to overcome the cognitive dissonance outlined and to exploit the benefits of SE more effectively. Although the conflict between revenue interests and gambler protection cannot be fully resolved, public health approaches including surveillance and enforcement of SE by an independent body rather than by the providers themselves are required.

## Data Availability

The datasets generated and/or analysed during the current study are not publicly available due to the qualitative nature of the study (amount of audio data and transcripts, availability only in German) but the transcripts of the interviews are available from the corresponding author on reasonable request.
